# Cherenkov Radiation Control via Self-accelerating Wave-packets

**DOI:** 10.1038/s41598-017-08705-4

**Published:** 2017-08-18

**Authors:** Yi Hu, Zhili Li, Benjamin Wetzel, Roberto Morandotti, Zhigang Chen, Jingjun Xu

**Affiliations:** 1 The Key Laboratory of Weak-Light Nonlinear Photonics, Ministry of Education, School of Physics and TEDA Institute of Applied Physics, Nankai University, Tianjin, 300071 P.R. China; 2 Institut National de la Recherche Scientifique, Université du Québec, Varennes, Québec J3X1S2 Canada; 3 School of Mathematical and Physical Sciences, University of Sussex, Falmer, Brighton BN1 9QH UK; 4 Institute of Fundamental and Frontier Sciences, University of Electronic Science and Technology of China, Chengdu, 610054 China; 5 National Research University of Information Technologies, Mechanics and Optics, St. Petersburg, Russia; 6 Department of Physics and Astronomy, San Francisco State University, San Francisco, California 94132 USA

## Abstract

Cherenkov radiation is a ubiquitous phenomenon in nature. It describes electromagnetic radiation from a charged particle moving in a medium with a uniform velocity larger than the phase velocity of light in the same medium. Such a picture is typically adopted in the investigation of traditional Cherenkov radiation as well as its counterparts in different branches of physics, including nonlinear optics, spintronics and plasmonics. In these cases, the radiation emitted spreads along a “cone”, making it impractical for most applications. Here, we employ a self-accelerating optical pump wave-packet to demonstrate controlled shaping of one type of generalized Cherenkov radiation - dispersive waves in optical fibers. We show that, by tuning the parameters of the wave-packet, the emitted waves can be judiciously compressed and focused at desired locations, paving the way to such control in any physical system.

## Introduction

**Figure 1:**
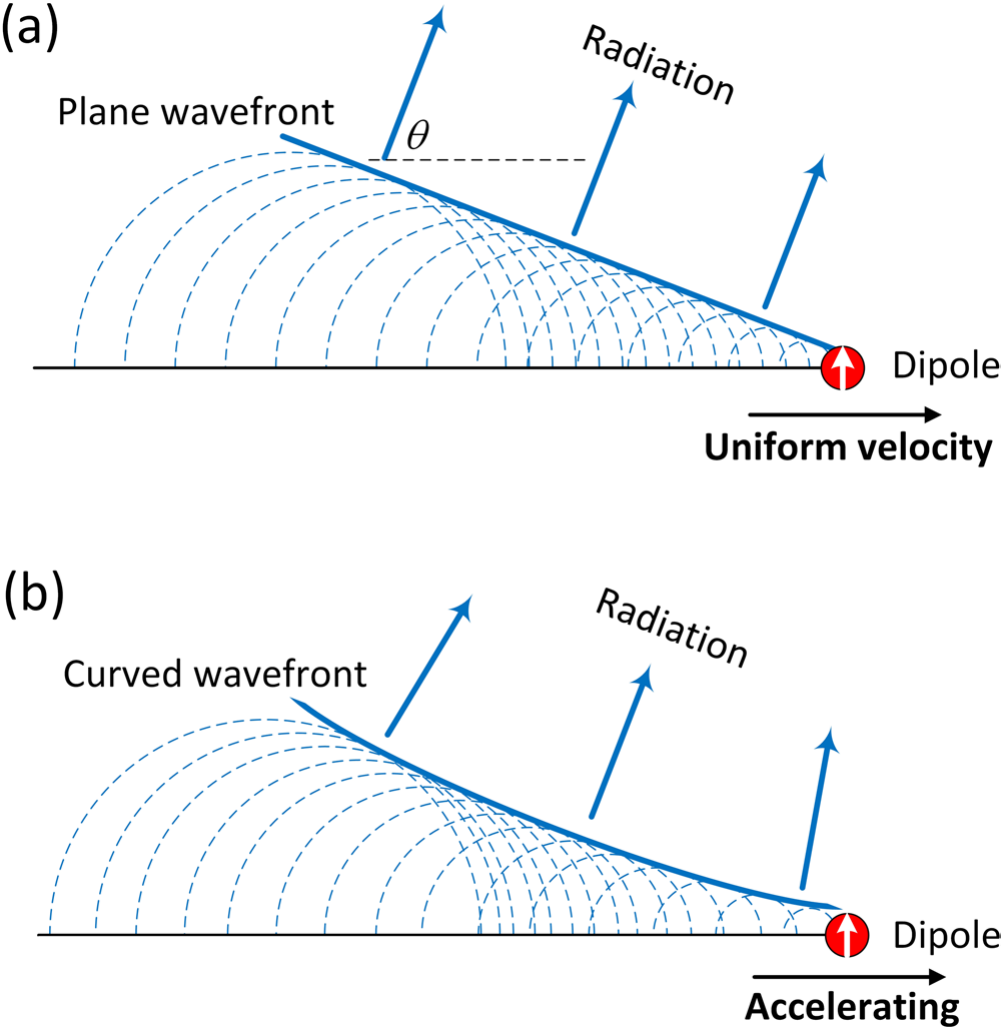
Schematic illustration of the process leading to the emission of Cherenkov radiation. The induced dipole moves with a uniform velocity (**a**), or in an accelerating fashion (**b**).

An underwater nuclear reactor is often surrounded by characteristic blue glows. The underlying principle associated with such a beautiful phenomenon is the longitudinal momentum conservation (or phase matching) between the dipoles induced in water by the moving charged particle (emitted from the nuclear reactor) and the electromagnetic radiation^1^. This traditional manifestation of Cherenkov radiation, well-known in the field of particle physics (Nobel Prize in 1958), is in fact a fundamental and universal phenomenon mediated by radiation from a moving source. Indeed, Cherenkov analogues appear in many fields, from boat waves and sonic booms in classic systems to emerging disciplines such as plasmonics^2^ or spintronic^3^. Particularly relevant examples can be found in nonlinear optics, including Cherenkov-type parametric processes^4^ and dispersive wave (DW) radiation in fibers optics^5^, where the role of the emitter is played by nonlinearly induced dipoles. In this framework, one of the predominant applications of this phenomenon is the realization of novel electromagnetic sources, which are nowadays extensively exploited in generating supercontinuum, Terahertz waves^6–8^ as well as on-chip frequency combs^9^. In these processes, the dipoles excited by, e.g., a particle or wave-packet moving in a medium at a high enough yet constant velocity, emit spherical waves (a single frequency is assumed) at each longitudinal position. The interference of these waves results in the formation of a radiation cone, in which the last excited dipole is always located at the apex (Fig. 1(a)). Note that the direction of the emitted radiation is given by the relation cos*θ* = *c*/(*nv*_*p*_), where *θ* is taken relatively to the particle motion, *c* is the speed of light in vacuum, *n* is the refractive index of the medium, and *v*_*p*_ is the velocity of the charged particle. For practical applications, additional optical diffractive/dispersive elements (such as lenses and prisms) are often needed to concentrate the radiation that is otherwise diverging. Alternatively, one may employ specially designed dielectric/metallic structures for shaping and steering the Cherenkov radiation^10–13^. These methods have their intrinsic drawbacks if limited space is an issue or dynamic control is also important.

While numerous works have targeted the effective control of Cherenkov radiation^10–13^, it is commonly believed that, if the induced dipole excitation moves at a constant velocity (Fig. 1(a)), such a goal is complex or even impossible to achieve in homogeneous media^11^ without using any external optical component. Yet, if the dipole excitation is accelerated (i.e., *v*_*p*_ is distance dependent), the direction in which the radiation is emitted (characterized by *θ*) varies along propagation, allowing for converging/diverging beams, as shown by a typical case plotted in Fig. 1(b) (here a single frequency is assumed for the sake of simplicity). However, in this case, the charged particle or the pump wave inducing the dipoles should also accelerate, often requiring the use of external forces and/or the realization of complex set-ups^14, 15^. Radiation shaping of this kind might have occurred in previous studies, but has neither been clearly observed (i.e., being typically concealed by other radiation dynamics) nor, to the best of our knowledge, been reported. This is probably due to the lack of a controllable acceleration associated to either charges or pumps.

A powerful alternative to all previous schemes is given by self-accelerating wave-packets, as established in this work. Self-accelerating wave-packets were first predicted^16^ in quantum mechanics by Berry and Balazs in 1979, and later introduced to optics^17, 18^ by Christodoulides and co-workers. In particular, by simply applying an appropriate phase modulation on a plane wave, it is possible to generate a self-accelerating optical beam (wave-packet) in either free space or homogeneous media without the need of any external force or nonlinear effect. Remarkably, such self-accelerating wave-packets are fundamentally general, as they were later realized in a variety of physical systems including, in addition to optics, (surface) plasmonics^19–21^, matter^22^, water^23^ and acoustic^24^ waves. The particle-like property of these wave-packets^25^, along with readily controllable acceleration characteristics achieved via suitable phase engineering^26–28^, bring about the possibility of shaping the Cherenkov radiation by tuning the optical wave-packet acceleration.

In this work, we introduce such a control through a meaningful example of Cherenkov-like radiation, i.e., the DW generated by a light pulse propagating in an optical fiber. In particular, we show the active control of Cherenkov radiation by employing a self-accelerating pump pulse, where the compression of the DW (temporally equivalent to spatial focusing) can be achieved by either tuning the pump pulse power or its temporal acceleration. Our theoretical predictions are in agreement with both numerical simulations and experimental measurements. Furthermore, we foresee that the concept developed here could be readily extended to any wave system beyond optics.

DWs are produced by an optical pulse propagating in a dispersive medium under the combined action of a Kerr nonlinearity and higher order dispersion terms^5^. Such a nonlinear frequency conversion process can be employed to realize tunable ultrashort pulse sources^29^ or to generate broadband supercontinuum^6^. In order to gain insight on the impact of the accelerating pulses in the DW dynamics, we start our analysis with a simplified model of pulse propagation in optical fibers, i.e. the nonlinear Schrödinger equation (NLSE). In this case, as we only include the Kerr nonlinearity and dispersion terms up to the 3^rd^ order, the equation takes the following normalized form (see Supplementary Information and ref. 30):

1 $$i\frac{\partial u}{\partial {\xi }}+\frac{1}{2}\frac{{\partial }^{2}u}{\partial {{\tau }}^{2}}-i{\sigma }\frac{{\partial }^{3}u}{\partial {{\tau }}^{3}}+{|u|}^{2}u=0.$$

where *ξ* (or *τ*) is the normalized distance (or time delay). *u*(*τ*, *ξ*) is the normalized envelope of the electric field moving in the time frame of the pump pulse central frequency, and ${\sigma }={{\beta }}_{3}/(6|{{\beta }}_{2}|{T}_{0})$. In the latter expression, *T*_0_ is an arbitrary time scale, while *β*_2_ (<0) and *β*_3_ represent the strength of the 2^nd^ and the 3^rd^ order dispersion, respectively. Assuming that the peak power of the pump pulse (expected to be the main contributor to the induced Kerr nonlinearity) follows a spatiotemporal path denoted by *τ*_*r*_ (i.e., a *ξ*-dependent time delay), its associated instantaneous frequency (denoted as *v*_*k*_) can be determined as follows:

2 $$\frac{d{{\tau }}_{r}({\xi })}{d{\xi }}={v}_{k}-3{\sigma }{v}_{k}^{2}$$

**Figure 2:**
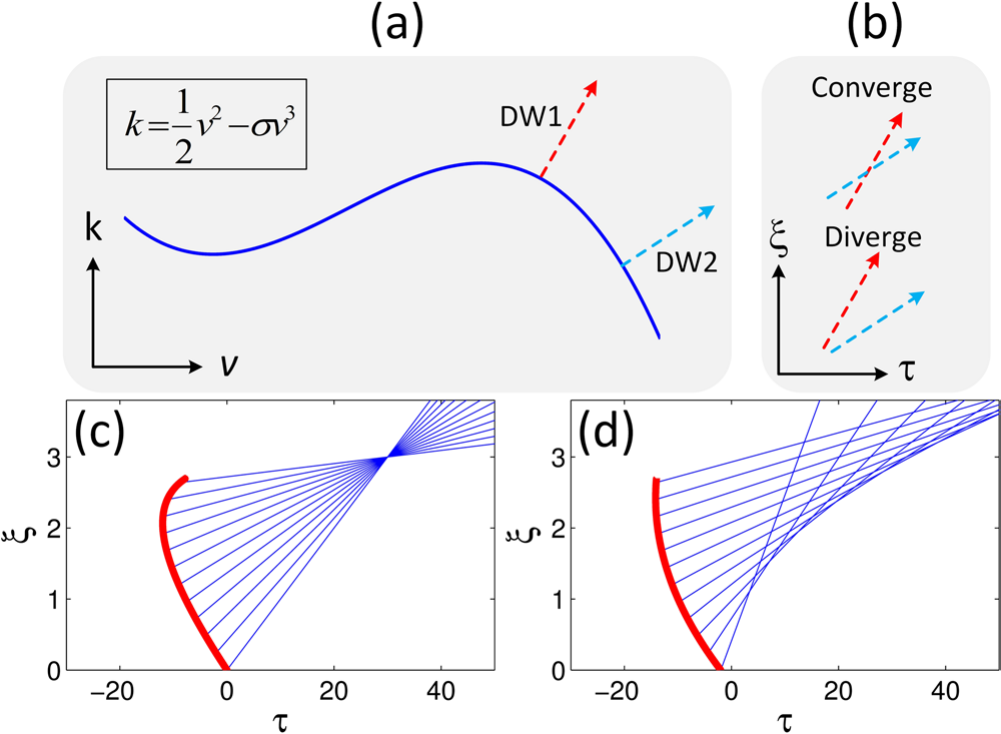
Description of the dynamical properties of dispersive waves (DWs) via ray tracing. (**a**) Sketch of a typical dispersion relationship defining the DW propagation direction. (**b**) Either divergence or convergence of the two dispersive waves shown in (**a**) can be achieved, depending on the point of origin of each individual DW. (**c**) and (**d**) show that the DWs (blue lines) can either focus to a point, or form a caustic, according to the trajectory followed by the pump pulses (red lines).

If the pump pulse accelerates, *v*_*k*_ is distance dependent, leading to the generation of DWs with different frequencies ${v}_{DW}=\frac{1}{2{\sigma }}-2{v}_{k}$, emitted at various propagation distances (see Supplementary Information for further details). This feature can be inferred from the dispersion relationship sketched in Fig. 2(a), for which the propagation direction (here defined as *dτ*/*dξ*) of the DWs generated at a given point on the curve (i.e. at a given frequency) can be obtained as the derivative −(*dk*/*dv*)^−1^, where *k* is the wave number and *v* is the angular frequency in normalized units. In this case, depending on the pump pulse trajectory, DWs with different frequencies are radiated at different times *τ* (and distances *ξ*), thus leading to either the convergence or divergence of the DWs, as illustrated in Fig. 2(a,b). In our analysis, we consider a typical case of single mode fiber propagation where the optical pump wavelength is located in the fiber anomalous dispersion regime (i.e. *β*_2_ < 0) and *β*_3_ is positive. Under these conditions, in order for the DWs to converge, the lower frequency components should be generated before the higher ones. In such a case, depending on the pump pulse acceleration, various scenarios of pulse shaping can be realized, including the “focusing” of the DWs into a single spatiotemporal location (Fig. 2(c)) or the formation of so-called “caustics”^31^ as illustrated in Fig. 2(d).

**Figure 3:**
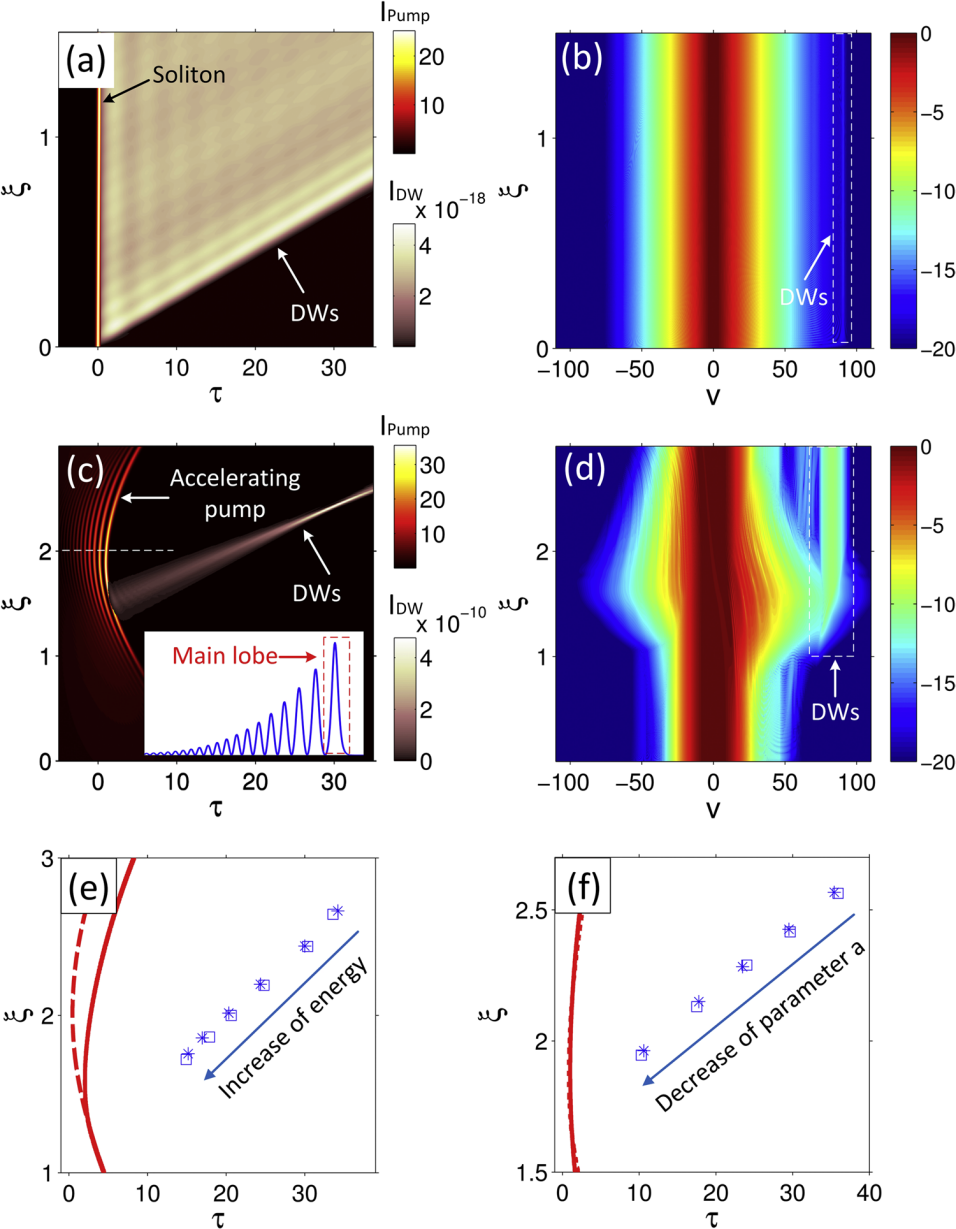
Generation and evolution of DWs when the pump pulse is either a soliton (Row 1) or an Airy pulse (Rows 2 and 3). (**a**,**c**) Numerical results for the temporal evolution of the pump pulse (hot-color) superimposed with the associated generation of DWs (in pink – retrieved by spectral filtering). Since the intensities of DWs are significantly lower than those of the pumps, different color scales are used for a better visualization. The inset in (**c**) depicts the temporal profile (featured by a main lobe) of the accelerating pulse at a selected distance (marked by the horizontal dashed line). (**b**,**d**) show the corresponding spectral intensity evolution (in a logarithmic scale). Note that the dashed rectangle highlights the generated DW spectral components. Row 3 illustrates the possibility to control the DW compression by linearly tuning either the normalized pulse energy from 20 to 120 (**e**), or the pump pulse acceleration (i.e., the value of the parameter *a*) from 0.024 to 0.032 (**f**). The locations of the DW converging points are calculated, for each case, from both theory (stars) and numerical simulations (squares). Correspondingly, the dashed (solid) lines represent the trajectory followed by the pulse main lobe for the minimum (maximum) input power in (**e**), or the maximum (minimum) acceleration in (**f**). Note that all the values are normalized.

In what follows, we aim at providing a proof of principle of versatile DW reshaping by employing accelerating pulses. For illustrative purposes, we first study the evolution of a fundamental soliton moving at constant velocity, as well as the DWs (exhibiting the feature of a plane wave) that are generated from such a pulse (see Fig. 3(a)). Not surprisingly and consistently with the properties of a plane wave, the corresponding DWs spectral components possess a narrow linewidth (generated around the frequency *v* = 90) as shown in Fig. 3(b).

In contrast, by using self-accelerating pump pulses, it is possible to generate “focusing” DWs through the scheme illustrated in Fig. 2(c), as we show here. As a typical example, we consider an Airy (one type of self-accelerating) pulse, which can be readily generated by applying a spectral cubic phase modulation^17^ via a dispersive system (such as a waveshaper, gratings and fibers). In Fig. 3(c,d), we present the evolution dynamics of an Airy pulse whose initial normalized spectrum at *ξ* = 0 is $\exp (-{v}^{2}/{{v}_{0}}^{2})\exp (ia{v}^{3}+ib{v}^{2})$ (see Supplementary Information). In such a regime, DWs with normalized frequencies in the range between *v* ≈ 70 and 90 are excited from the main lobe of the accelerating pump, for which the lower DWs frequency components appear prior to the higher ones. As expected in this case, the DWs converge (Fig. 3(c)). Different DW focusing dynamics can be reached by further tuning the accelerating pump pulse parameters. For example, by varying the energy of the accelerating pulse, it is possible to alter the path of the main lobe as a consequence of self-phase modulation. In turn, this results in higher peak powers at the propagation onset, a more pronounced curvature of the pump trajectory and eventually a stronger focusing of the DWs. Numerical results illustrating this approach are presented in Fig. 3(e), in which we plot the spatio-temporal location of the DWs focusing as a function of the input pulse energy (blue squares). As can be seen, depending on the input energy, the trajectory of the pulse (red lines) is significantly modified, thus yielding to a stronger focusing of the DWs at higher energies. Such a control on the DWs focusing can also be performed by directly modifying the curvature of the pulse trajectory, i.e. changing the modulation depth (given by the parameter *a*) of the Airy spectral cubic phase. A smaller parameter *a* corresponds to a larger bending of the main lobe trajectory, thus leading to a faster DW focusing (Fig. 3(f)). For both degrees of freedom, the converging points are almost collinear, and the “focal length” exhibits a quasi linear dependence on the parameter *a*, together with a nonlinear trend as a function of the input pulse energy. By considering the path followed by the peak power of the pump, it is possible to directly retrieve, analytically, the spatio-temporal location where the DWs compress to a minimum width (see Supplementary Information). Such analytic results are marked by the blue stars in Fig. 3(e,f), showing good agreement with the corresponding numerical simulations (blue squares). However, we should note that alternative effects such as intra-pulse Raman scattering^32^ or soliton collision can also lead to pulse acceleration (either positive or negative). Nevertheless, these effects are usually difficult to control. This clearly limits the tunability of the spatiotemporal trajectory of the pump pulse, and ultimately hinders the observation of focusing (or active shaping) of the DWs via accelerating pumps (which indeed has not been experimentally realized so far). In our approach, the acceleration of the pump pulses comes directly from the initial spectral phase modulation imprinted on the input pulse rather than from any external effect that could lead to drastic experimental limitations. In particular, if we consider the example in ref. 33, we find that the power and the acceleration (negative) of fundamental solitons under the effect of intra-pulse Raman scattering are deterministically fixed by the system parameters. It is indeed worth underlying, once more, that the focusing of the DWs reported here is simply obtained by adjusting the pump pulse’s spatiotemporal trajectory, whose parameters can be controlled by using the methods shown in ref. 26–28 (thus offering a versatile tool to efficiently shape Cherenkov radiation). We also note that our approach is qualitatively different from (even though may be seemingly resembling to) the so-called “optical event horizons” recently reported in the literature. In the latter case, DW management is achieved subsequently to their generation^34–36^, being typically observed via a controlled reflection of a weak probe pulse onto a more intense pump pulse, for which both relative delay and propagation parameters need to be properly adjusted.

In order to verify the effectiveness of our scheme in shaping the DWs, we perform experiments using accelerating pulses launched into a dispersion shifted fiber (DSF), whose zero dispersion wavelength is located at 1547.7 nm. An Airy pulse is generated by applying a cubic phase modulation, spectrally encoded via a pulse shaper on a transform-limited pulse that is emitted by a passive mode-locked femtosecond laser, and then injected into a 3.9-km-long DSF (see Supplementary Information). At the output of this fiber, where the DWs are generated, the accelerating pump is filtered out by means of a tunable filter. The residual DW frequency components are then analyzed with either an optical spectral analyzer (OSA) or an intensity autocorrelator working together with an erbium-doped fiber amplifier (EDFA) (see Supplementary Information for details). To evaluate the DW longitudinal evolution, two additional DSF sections of different lengths (1.4 km and 2.8 km) are connected to the filter output. We emphasize that the EDFA is maintained at a low amplification level to avoid the reshaping of the DWs potentially caused by self-phase modulation.

**Figure 4:**
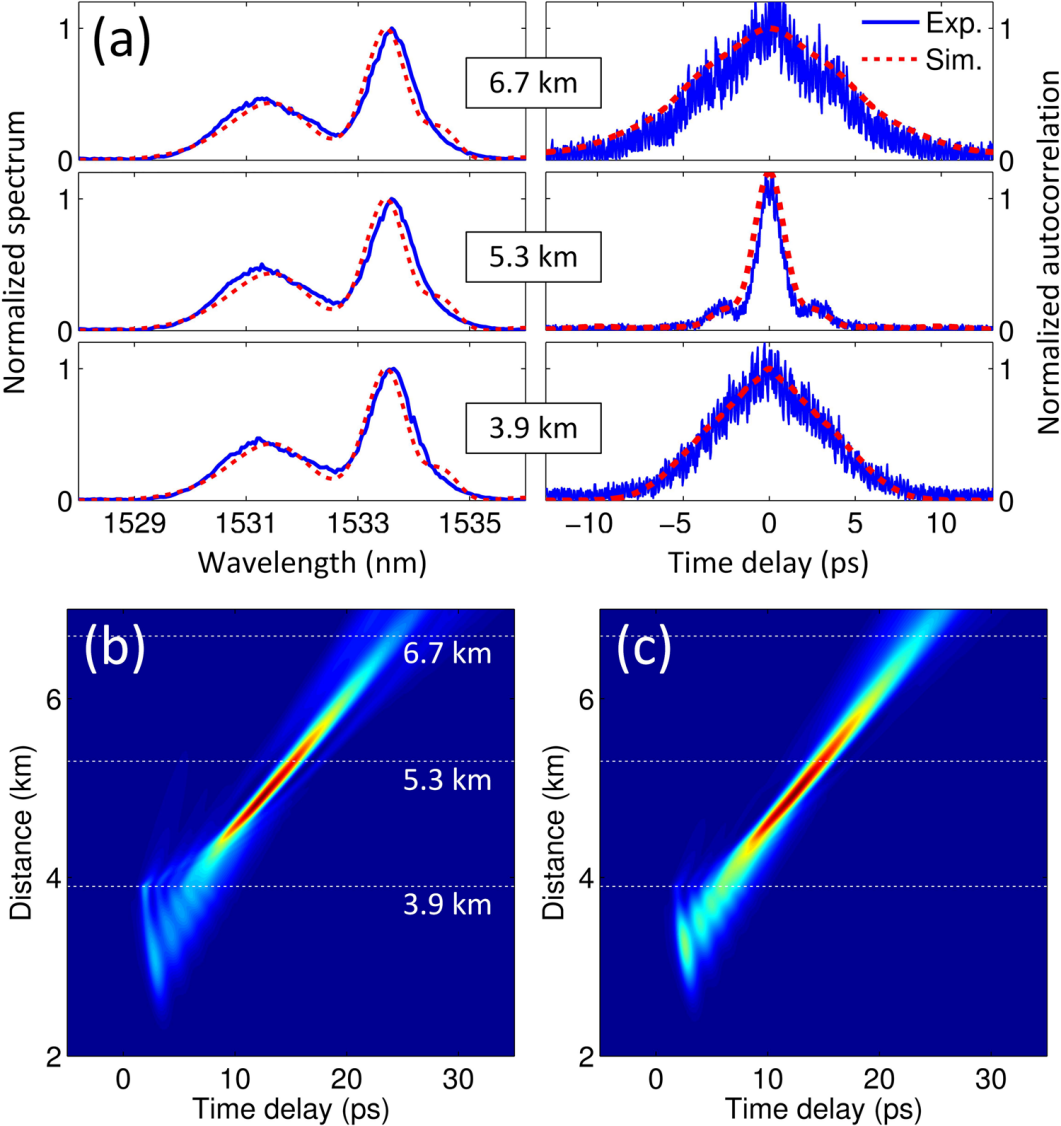
Observation and the associated simulations of DW shaping by employing a self-accelerating pulse. (**a**) Measured spectrum (left column) and autocorrelation intensity (right column) of the generated DWs at the output for different lengths of DSF. (**b**) Simulated DW evolution obtained by solving equation (1) with the parameters fitting the experimental data. (**c**) Same as (**b**) using a generalized model which includes the additional effects of Raman scattering, self-steepening and attenuation. We note the marginal difference in the DW dynamics following the inclusion of higher order parameters (see Supplementary Information). The white dashed lines in (**b**) and (**c**) mark the three fiber output lengths used in (**a**) for the comparison between our experiments and the related simulations based on the NLSE.

Using an initial average power of 270 μW and a spectral phase modulation given by *Aω* ^3^ + *Bω* ^2^, where *A* = 0.5 ps^3^, *B* = −3.3 ps^2^ (such a value can prevent the Airy pulse from forming solitons at the propagation onset while complying, at the same time, with the limited resolution of our waveshaper), and *ω* is the angular frequency, the measured spectra and autocorrelation traces at the three distances of interest are summarized in Fig. 4(a). As seen from these figures, the DWs are generated in the normal dispersion region spanning from 1529 nm to 1536 nm, whose spectrum is featured by two noticeable peaks. Once the pump is filtered out (at z = 3.9 km), the DW spectrum does not change significantly over subsequent propagation (indicating either the absence or the negligible impact of nonlinear effects - i.e. self-phase modulation- in the DWs focusing process). On the other hand, in the temporal domain, one can see a clear compression of the DWs. In particular, the width of the autocorrelation trace shrinks to a minimum at z = 5.3 km. At this point of DWs compression, we can notice two small side peaks on the autocorrelation trace, suggesting that the trajectory of the accelerating pulse does not match perfectly the path shown in Fig. 2(c). Indeed, it seems to rather follow a trajectory analogous to the caustic case shown in Fig. 2(d). Nevertheless, our experiments confirm that the DW compression is associated to the accelerating motion of the pump pulse. The generation and the evolution of the DWs can be visualized in Fig. 4(b), obtained by numerically solving equation (1). Here, as we did in our experiments, the pump pulse is discarded at z = 3.9 km. To assess the impact of a more realistic propagation, we have also performed numerical simulations based on a generalized NLSE model (see Supplementary Information)^6^, taking into account attenuation (0.2 dB/km) as well as higher nonlinear effects (i.e. Raman scattering and self-steepening). The corresponding numerical results are shown in Fig. 4(c), which reveal a very good agreement with both experimental results and NLSE-based simulations.

**Figure 5:**
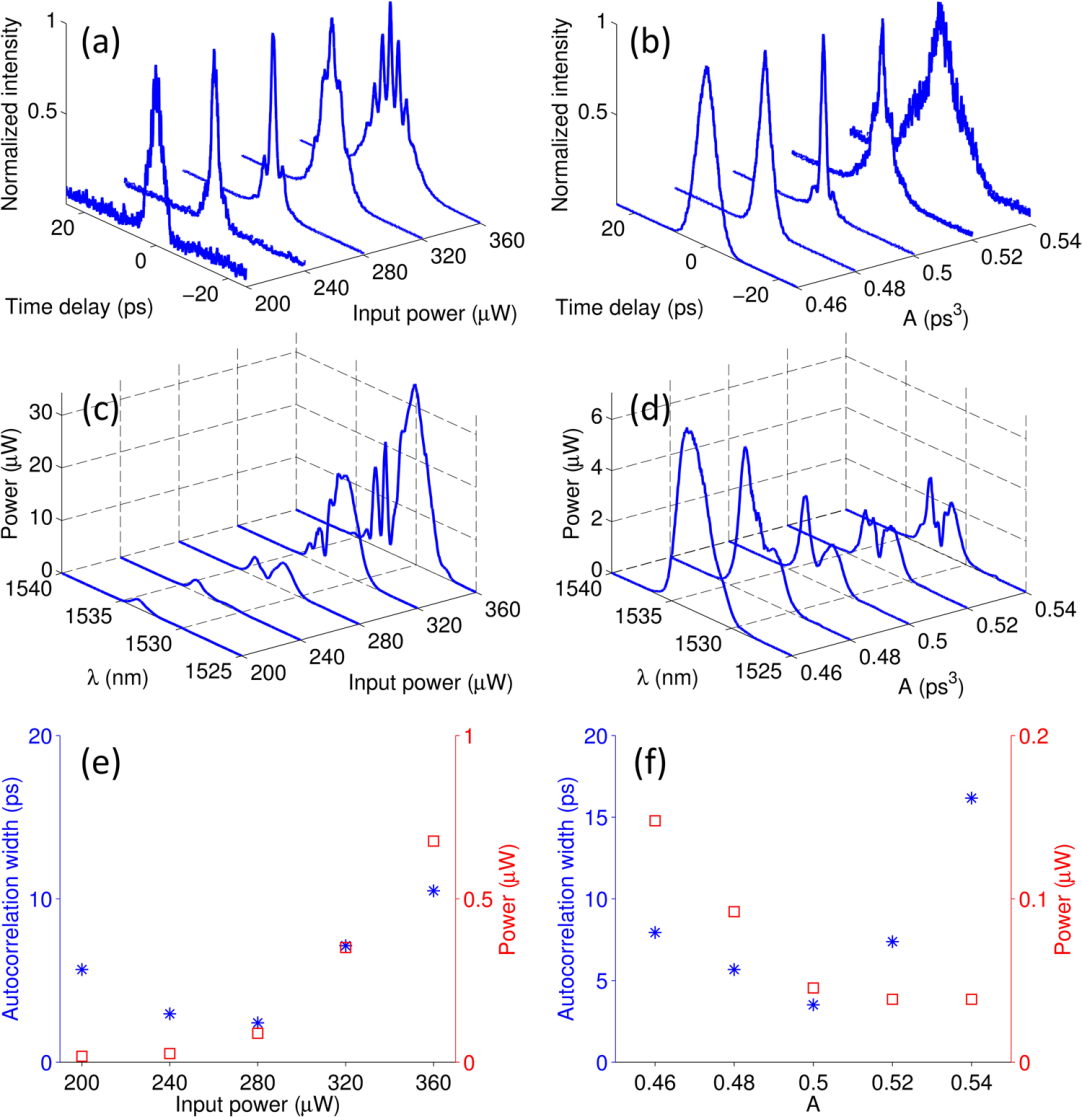
Experimental results of DW control using different input powers (left column) and depths of cubic phase modulation *A* (right column). The top and middle rows show the measured autocorrelation traces and spectra, respectively. In (**c**), the power of the DW spectrum becomes larger as a result of an enhanced main lobe compression of the Airy pulse - caused by a higher pump power. In (**d**), larger *A values* yield to a decreased peak power of the Airy pump, which leads in turn to reduced DWs powers. (**e**) and (**f**) correspond to (**a**,**c**) and **(b**,**d**), respectively. Here, both the width of the autocorrelation trace (measured at the *e* ^−1^ level of the maximum intensity) and the corresponding DWs powers extracted from the spectral measurements are plotted.

As illustrated in Fig. 3, we expect to control the DW focusing by either adjusting the input power or the acceleration rate of the pump pulse. In Fig. 5, we summarize the experimental results obtained by adjusting these control parameters for an Airy pulse propagation in a 5.3-km-long DSF. In a first experiment, we keep the cubic phase constant (i.e., *A* = 0.5 ps^3^), while adjusting the input power from 200 μW to 360 μW (Fig. 5(a) and (c)). As shown in Fig. 5(a), the width of the DW autocorrelation traces initially exhibits a decrease (compression), followed by an increase (expansion) as the pump input power is tuned up. An optimal DW compression is observed at a power of 260 μW (Fig. 5(a)). In a subsequent experiment, we fix the power at this optimal level, while changing the initial cubic phase (i.e., the parameter *A*). Also in this case, we observe the compression/expansion of the DW autocorrelation traces, thus attesting that the parameters previously used in Fig. 4 are close to the optimal values for achieving focusing at z = 5.3 km. The experimental results reported in Fig. 5 closely follow the numerical predictions of Fig. 3(e,f). Interestingly, although both the input power and the acceleration rate can be efficiently used to control the compression of DWs, the spectra associated to the two different tuning parameters (power and cubic phase) exhibit significant differences (see Fig. 5(c–f)). Modifications of the input power affect the bandwidth and power of the DWs more significantly than the changes in the pulse cubic phase. This can be readily understood by recalling that large input powers are associated with enhanced temporal compressions of the accelerating pulse main lobe. This in turn leads to the excitation of DWs featured by a wider spectral bandwidth and a larger power as illustrated in Fig. 5(c) and (e).

In this work, we demonstrate, in a fiber optics framework, effective control of the generation and evolution of DWs, a specific yet powerful example of Cherenkov emission. Although we only studied a particular type of temporal self-accelerating pulses (Airy pulses) propagating in the anomalous dispersion regime of a fiber, our approach can be readily extended, in principle, to the case of more arbitrary “bending” wave-packets and to the normal dispersive regime^37, 38^. Consequently, our results may find widespread applications in developing/improving efficient optical sources, in the optimization of supercontinuum generation and in controlling light with light^39, 40^. From a more fundamental point of view, further degrees of control are expected in 3D (spatio-temporal) and/or nonlinear systems^41–43^. Moreover, the proposed idea can be translated from optics to several other systems in nature that may lead to new exciting applications. For example, it may be possible to concentrate the energy of the acoustic shock-waves generated by a supersonic object on a specific target, or to increase the sensitivity of the state-of-the-art detectors used in today’s nuclear physics experiments.

## Electronic supplementary material

Supplementary information
