# Supplementary material for: Cherenkov Radiation Control via Self-accelerating Wave-packets
Source: Sci Rep. 2017 Aug 18;7:8695. doi: 10.1038/s41598-017-08705-4 (PMC5562856; doi:10.1038/s41598-017-08705-4)
Supplement: Supplementary file 1 — Supplementary information [file 41598_2017_8705_MOESM1_ESM.doc]

**Cherenkov Radiation Control via Self-accelerating Wave-packets**

Yi Hu, Zhili Li, Benjamin Wetzel, Roberto Morandotti, Zhigang Chen, Jingjun Xu

Supplementary information:

***Theory:***

In the time frame moving with the pump pulse central frequency (denoted as *ω_p_*), the propagation equation takes the form of the nonlinear Schrödinger equation:

(3)

where *ϕ* is the envelope of the electric field, *β*_2_ and *β*_3_ are the 2^nd^ and the 3^rd^ order dispersion coefficients, respectively, *γ* is the nonlinear coefficient, *z* is the distance, and *T* is the time delay. By employing the following transformation:

, (4)

where *T*_0_ is an arbitrary time scale, equation (3) takes the normalized form as described by equation (1), i.e.,

$i\frac{\partial u}{\partial\xi}-\frac{\beta_{2}^{'}}{2}\frac{\partial^{2}u}{\partial\tau^{2}}-\frac{i\beta_{3}^{'}}{6}\frac{\partial^{3}u}{\partial\tau^{3}}+\left|u\right|^{2}u=0,$ (5)

where$\beta_{2}^{'}=-1$and$\beta_{3}^{'}=6\sigma$are the normalized 2^nd^ and the 3^rd^ order dispersion coefficients, respectively. The linear dispersion relation is obtained by neglecting the nonlinear term and substituting $u=\exp\left[i\left(-k\xi-v\tau\right)\right]$into equation (5), where *k* is the normalized wave number and *v* is the normalized angular frequency, so that:

$k=-\frac{1}{2}\beta_{2}^{'}v^{2}-\frac{1}{6}\beta_{3}^{'}v^{3}$. (6)

Dispersive waves (DWs) are excited by nonlinear frequency conversion, so that each single DW frequency *v* is set by the resonance condition^35^. If we first consider the center frequency (*v_p_* = 0) of the pump, the phase matching condition$k=k_{p}$^35^, where $k_{p}=k\left(v_{p}=0\right)=0$ is the pump wave vector, is then satisfied for:

$-\frac{1}{2}\beta_{2}^{'}v^{2}-\frac{1}{6}\beta_{3}^{'}v^{3}=0$. (7)

Using a process similar to the derivation of equation (7), one can readily get the formula for calculating the DW frequency ($v_{DW}$) associated with one (*v_k_*) of the pump frequency by employing that the dispersion associated with *v_k_* is described as $\beta_{2}^{'}\left(v_{k}\right)=6\sigma v_{k}-1$(derived according to the formula^30^ $d\beta_{2}^{'}/dv=\beta_{3}^{'}$) and$\beta_{3}^{'}\left(v_{k}\right)=6\sigma$($\beta_{3}^{'}$is a constant since the dispersion higher than the 3^rd^ order one is not under consideration):

$-\frac{1}{2}\left(6\sigma v_{k}-1\right)\left(v_{DW}-v_{k}\right)^{2}-\sigma\left(v_{DW}-v_{k}\right)^{3}=0$. (8)

We then obtain:

$v_{DW}=\frac{1}{2\sigma}-2v_{k}$. (9)

For a self-accelerating pulse, such an offset$v_{k}$is in fact distance dependent. Assuming that the pump pulse propagates following a spatiotemporal trajectory given by *τ*=*τ_r_*(*ξ*), the tangent to this curve can be determined via the linear dispersion relationship as:

$\frac{d\tau_{r}\left(\xi\right)}{d\xi}=-\frac{dk}{dv}=-v+3\sigma v^{2}$. (10)

In this framework, the distance dependent frequency offset is given by:

$v_{k}=\frac{1-\sqrt{1+12\sigma\frac{d\tau_{r}\left(\xi\right)}{d\xi}}}{6\sigma}$ . (11)

Note that the other solution of equation (10) is invalid for the frequency offset of the pump. By substituting equation (11) into equation (9), one can derive the spatio-temporal evolution of each dispersive wave spectral component, all described by straight lines:

$T=sz$, (12)

where $s=-v_{DW}+3\sigma v_{DW}^{2}$. These lines, under appropriate initial conditions, can be forced to assume a desired distribution. For instance, the various DW components can be set in such a way that they converge towards a unique point, or form a caustic. One can then obtain the acceleration configuration of the pump pulse by solving equation (9), equation (11) and equation (12).

***NLSE Simulations:*** Our numerical results are obtained by solving the NLSE given in equation (1). For illustrative purposes, and to highlight the universality of our approach, we start our analysis using a value of *σ* different from the one imposed by our experimental constraints. Indeed, for the simulations in Fig. 2 and Fig. 3, *σ* is set to 1/180 (so that the frequency components for the generated DWs are centered at ~ 90 according to equation (9)). Here, the fundamental soliton has the form $u(\tau,\xi=0)=\eta\mathrm{sech}(\eta\tau)$ (where *η*=5), while the Airy pulse under investigation has an input spectrum proportional to $\exp(-v^{2}/v_{0}^{2})\exp(iav^{3}+ibv^{2})$ with parameters *v*_0_=8.5, *a*=0.03, and *b*=-1.1. Since *T*_0_ can be arbitrarily chosen, the simulations carried out using the above parameters correspond in fact to various realistic cases, which can be found by properly choosing the fiber dispersion and the pulse parameters. For instance, by selecting a time scale *T*_0_ = 1 ps, the ratio of the dispersion coefficient has to be $\beta_{3}/\left|\beta_{2}\right|$ = 1/30 ps in order to give realistic propagation conditions for a value of *σ* = 1/180. In such a case, easily obtained by judiciously tuning the pump pulse wavelength in a given optical fiber, the full width at half-maximum (FWHM) pulse duration for the soliton is ~350 fs, while the FWHM bandwidth of the Airy pulse is ~13 nm for the cases respectively illustrated in Fig. 3.

***GNLSE Simulations:*** For a more accurate modeling of the optical pulse propagation in our experiments, we also provide numerical simulations based on the generalized nonlinear Schrödinger equation (GNLSE)^6^, including the impact of attenuation and higher order effects on the pulse evolution:

(13)

In equation (13), *α* = *α_dB_*log(10)/10 km^-1^, where *α_dB_* is the power loss coefficient, *γ* is the nonlinear coefficient, and *τ_sh_* =1/*ω*_0_ is the shock coefficient responsible for self-steepening (being *ω*_0_ the center angular frequency). Furthermore, the nonlinear response function $R\left(t\right)=\left(1-f_{R}\right)\delta\left(t\right)+f_{R}h_{R}\left(t\right)$includes both instantaneous electronic Kerr and delayed Raman contributions, where$f_{R}$= 0.18 represents the fraction of the overall nonlinear response due to Raman scattering. Here, $h_{R}(T)$is approximated by the expression$\exp\left(-T/\tau_{2}\right)\sin\left(T/\tau_{1}\right)\left[\left(\tau_{1}^{2}+\tau_{2}^{2}\right)/\tau_{1}\tau_{2}^{2}\right]$ (*τ*_1_=12.2 *fs* and *τ*_2_=32 *fs*) for *T* > 0, and *h_R_* = 0 for *T* < 0. The other parameters in equation (13) have the same definition as those in equation (3).

In our simulations, aimed at reproducing our experimental results, we used the following DSF parameters: the fiber dispersion coefficients are *β*_2_ = -9.0e-28 s^2^/m, and *β*_3_ = 1.3e-40 s^3^/m, respectively, the nonlinear coefficient is *γ* = 0.49 W^-1^km^-1^ and the losses are estimated to be *α_dB_* = 0.2 dB/km. Note that, by neglecting higher order effects, i.e. setting *α*, *τ_sh_* and$\gamma_{R}$to zero, equation (13) can be reduced to equation (3) - the NLSE equation previously described.

***Experiments:*** To perform our measurements, we used a passively mode-locked laser from Pritel (10 MHz repetition rate), emitting transform-limited hyperbolic secant pulses featured by a central wavelength of ~1557 nm and a corresponding bandwidth of ~3 nm. Such pulses are transformed into Airy waves by employing a pulse shaper (Finisar, 4000S). A tunable filter (Santec, OTF-350), employed to eliminate the pump, is set to transmit wavelengths ranging from 1527 nm to 1537 nm. An EDFA (also from Pritel) was used to amplify the DW before its temporal characterization by means of an intensity autocorrelator. Such an EDFA can operate on pulses of 300 fs (or longer) without inducing significant pulse broadening. Additionally, self-phase modulation in the pulse characterization process was avoided by carefully checking that the autocorrelation trace remained unchanged for various amplification levels, at sufficiently low values of the current pumping the EDFA.
